# The Effects of Tocotrienol and Lovastatin Co-Supplementation on Bone Dynamic Histomorphometry and Bone Morphogenetic Protein-2 Expression in Rats with Estrogen Deficiency

**DOI:** 10.3390/nu9020143

**Published:** 2017-02-15

**Authors:** Kok-Yong Chin, Saif Abdul-Majeed, Norazlina Mohamed, Soelaiman Ima-Nirwana

**Affiliations:** 1 Department of Pharmacology, Universiti Kebangsaan Malaysia Medical Centre, Cheras 56000, Malaysia; chinkokyong@ppukm.ukm.edu.my (K.-Y.C.); azlina@ppukm.ukm.edu.my (N.M.); 2 Department of Anatomy and Cell Biology, University of Pennsylvania, PA 19104, USA; saif_saad83@yahoo.com

**Keywords:** calcium, mineralization, menopause, mevalonate, osteopenia, osteoporosis, vitamin E

## Abstract

Both tocotrienol and statins are suppressors of the mevalonate pathway. Supplementation of tocotrienol among statin users could potentially protect them against osteoporosis. This study aimed to compare the effects of tocotrienol and lovastatin co-supplementation with individual treatments on bone dynamic histomorphometric indices and bone morphogenetic protein-2 (BMP-2) gene expression in ovariectomized rats. Forty-eight female Sprague-Dawley rats were randomized equally into six groups. The baseline was sacrificed upon receipt. All other groups were ovariectomized, except for the sham group. The ovariectomized groups were administered orally daily with (1) lovastatin 11 mg/kg/day alone; (2) tocotrienol derived from annatto bean (annatto tocotrienol) 60 mg/kg/day alone; (3) lovastatin 11 mg/kg/day, and annatto tocotrienol 60 mg/kg/day. The sham and ovariectomized control groups were treated with equal volume of vehicle. After eight weeks of treatment, the rats were sacrificed. Their bones were harvested for bone dynamic histomorphometry and BMP-2 gene expression. Rats supplemented with annatto tocotrienol and lovastatin concurrently demonstrated significantly lower single-labeled surface, but increased double-labeled surface, mineralizing surface, mineral apposition rate and bone formation rate compared to individual treatments (*p* < 0.05). There was a parallel increase in BMP-2 gene expression in the rats receiving combined treatment (*p* < 0.05). The combination of annatto tocotrienol and lovastatin exerted either additively or synergistically on selected bone parameters. In conclusion, tocotrienol can augment the bone formation and mineralization in rats receiving low-dose statins. Supplementation of tocotrienol in statin users can potentially protect them from osteoporosis.

## 1. Introduction

Hypercholesterolemia is a prevalent condition among middle-aged and elderly populations worldwide [1,2,3]. Statins are the most commonly prescribed medication for the treatment of this condition to prevent cardiovascular disease [4]. The middle-aged and elderly populations are also susceptible to osteoporosis. It is a condition characterized by degeneration of bone mass and deterioration of skeletal microarchitecture, leading to decreased bone strength and increased risk of fracture [5]. Post-menopausal women are particularly susceptible to osteoporosis because rapid bone loss occurs after the cessation of ovarian estrogen production [6]. 

Meta-analyses have concluded that statins could increase bone mineral density of its users and protect them from osteoporosis [7,8,9]. This pleiotropic effect of statins on bone is mediated through the suppression of the mevalonate pathway, which plays an integral part in both cholesterol synthesis and bone metabolism. The inhibition of 3-hydroxy-3-methyl-glutaryl-coenzyme A reductase (HMGCR) and the subsequent reduction in isoprenoid synthesis tilt the bone remodeling process in favor of formation over resorption [10]. However, most animal studies indicated that statins at doses higher than the clinical hypocholesterolemia regimen are required to exploit its bone-protective potential [11,12]. High-dose statins are often accompanied by adverse side-effects, such as myopathy, rhabdomyolysis, increased circulating transaminase level and risk of diabetes mellitus [13]. Thus, it is not a safe osteoporosis treatment option. 

Tocotrienol, a member of vitamin E family in addition to tocopherol, has been shown to exhibit bone protective action in various animal bone loss models [14,15,16]. Homologues of tocotrienol, namely alpha-, beta-, gamma-, and delta-tocotrienol, are available in mixtures derived from plant sources [17,18]. Oil derived from palm kernel, annatto seed and rice bran is rich in tocotrienol [19,20,21]. The skeletal protective actions of palm tocotrienol mixtures in post-menopausal animal model have been studied extensively [22,23,24,25]. Recent evidence also suggested that annatto tocotrienol supplementation at 60 mg/kg/day for eight weeks could prevent post-menopausal bone loss in rats by preserving the integrity of trabecular structure, increasing the number of osteoblast (bone forming cells), decreasing the number of osteoclast (bone resorbing cells) and maintaining bone biomechanical strength [26,27]. Annatto tocotrienol at 60 mg/kg/day also exerted strong skeletal anabolic effects in rats with testosterone deficiency by increasing the expression of bone formation genes coding for alkaline phosphatase (ALPL), beta-catenin (CTNNB1), collagen type I alpha 1 (COL1A1) and osteopontin (SPP1) [28]. A study by Deng et al. suggested that the bone protective activity of tocotrienol was mediated by the mevalonate pathway [29].

Taking all evidence into consideration, tocotrienol can potentially enhance the bone protective effects of statins among its users. Two previous reports indicated that concurrent supplementation of lovastatin at normal hypocholesterolemic dosage (11 mg/kg/day) and annatto tocotrienol at 60 mg/kg/day body weight prevented the degeneration of trabecular structure and bone strength in ovariectomized rats [26,27]. Lovastatin, alone, failed to do the same within the same treatment period [26,27]. However, the effects of lovastatin and annatto tocotrienol co-supplementation on bone formation and mineralization activity, as indicated by dynamic histomorphometric parameters, in ovariectomized rats have not been explored. The dynamic histomorphometry utilizes fluorescent labeling agents to visualize mineral deposition and formation activity in bone [30]. There is also no literature on the effects of the combined treatment on BMP-2, an integral bone formation signal that bridges mevalonate pathway and osteoblastic differentiation [31]. 

This study is a continuation of our previous studies [26,27] and aimed to compare the effects of lovastatin, annatto tocotrienol and the combination of both agents on bone dynamic parameters and skeletal BMP-2 mRNA expression in ovariectomized rats. We hypothesized that the combined treatment would result in better bone mineralization and formation in rats compared to individual treatments. This would be brought about by an increased skeletal BMP-2 expression. This study will complement our earlier attempts and establish tocotrienol as a bone protective agent for post-menopausal women at risk of both osteoporosis and hypercholesterolemia.

## 2. Materials and Methods 

### 2.1. Preparation of Annatto Tocotrienol and Lovastatin

Annatto tocotrienol containing 90% delta-tocotrienol and 10% gamma-tocotrienol was a gift from American River Nutrition (Hadley, MA, USA). This mixture was chosen because previous studies showed that tocotrienol mixture with less alpha-tocopherol was more effective in suppressing the activity of HMGCR [32]. In addition, gamma- and delta-tocotrienol were shown to be more effective compared to other isomers in lowering cholesterol level [33]. It was diluted 10 times in olive oil (Bartolini Emilio, Arrone Terni, Italy). Mevacor tablets (Merck, NJ, USA) containing 40 mg lovastatin was grounded and suspended in 0.5% carboxymethycellulose (Sigma-Aldrich, St. Louis, MO, USA). 

### 2.2. Animal Treatment

The study protocol was reviewed and approved by Universiti Kebangsaan Malaysia Animal Ethics Committee. A total of 48 three-month-old Sprague-Dawley female rats weighing 200–250 g were obtained from the Laboratory Animal Resource Unit, Universiti Kebangsaan Malaysia (Kuala Lumpur, Malaysia). They were housed in the animal laboratory of the Department of Pharmacology, Universiti Kebangsaan Malaysia Medical Centre (Kuala Lumpur, Malaysia) under standard conditions (27 °C; ambient humidity; natural dark light cycle; standard rat chow, and tap water ad libitum). After one week of acclimatization, they were randomly divided into six groups: baseline (BL), sham (SH), ovariectomized control (OVX), ovariectomized and treated with lovastatin (OVX+LOV), ovariectomized and treated with annatto tocotrienol (OVX+AnTT), ovariectomized and treated with lovastatin and annatto tocotrienol (OVX+LOV+AnTT). The BL group was sacrificed upon receipt. All groups except the SH underwent bilateral ovariectomy. The SH group was subjected to similar surgical stress but the ovaries were not removed. Treatment was initiated one week after ovariectomy to allow the rats to recuperate. The OVX+LOV and OVX+LOV+AnTT group received daily oral administration of lovastation (11 mg/kg/day) while the other groups received equal volume of 0.5% carboxymethylcellulose as vehicle. Annatto tocotrienol at 60 mg/kg body weight was administered daily orally to the OVX+AnTT and OVX+LOV+AnTT group while the other groups was given equal volume of olive oil as vehicle. All treatments regimens were administered using an 18 gauge oral gavage needle with round end when the animals were restrained. The rats were sacrificed after eight weeks of treatment by anesthetic overdose. Left and right femoral and tibial bones were harvested for analysis. 

### 2.3. Preparation of Bone Sample

The rats were administered calcein (Sigma-Aldrich, St. Louis, MO, USA) at 20 mg/kg body weight nine days and two days prior to euthanasia. Calcein is a fluorescent chromophore, which binds specifically to the skeleton, allowing direct visualization of mineralization. The left femurs was harvested, sectioned into halves sagittally, and fixed using alcohol. Next, the undecalcified bone was infiltrated and embedded using methyl methacrylate resin (Osteo-bed bone embedding kit, Polyscience, Warrington, PA, USA). The resin block was sectioned at thickness of 8 µm using a microtome (Leica, Wetzlar, Germany). 

### 2.4. Assessment of Dynamic Histomorphometric Indices 

The unstained slides were observed using a fluorescence microscope (Nikon Eclipse 80i, Tokyo, Japan). The secondary spongiosa in the metaphyseal region located 3–7 mm from the lowest point of growth plate and 1 mm from the cortical wall was sampled. The calcein-labeled surface of trabecular bone was measured manually using a Weibel grid with the aid of an image analyzer (MediaCybernetics Image Pro-Plus, Rockville, MD, USA). The dynamic histomorphometric parameters measured included single- (sLS/BS) and double-labeled surface (dLS/BS), mineralizing surface (MS/BS; extent of bone surface actively mineralizing), mineral apposition rate (MAR; distance between two labels in a double-labelled surface divided by the time between two calcein injections) and bone formation rate (BFR; the product of MAR multiplied by the fraction of labelled bone surface). 

### 2.5. Determination of Bone Morphogenetics Protein-2 (BMP-2) Expression in Bone

Approximately 40 g of bone tissue sampled from proximal tibial metaphyseal region was homogenized and RNA was extracted using RNeasy Lipid Tissue Mini Kit (QIAGEN, Venlo, The Netherlands). Concentration and purity of RNA was determined using the Nanodrop 2000 device (Thermo Fisher Scientific, Waltham, MA, USA). The real-time PCR reaction mixture was prepared using iScript One-Step RT-PCR reagent with SYBR Green (Bio-Rad, Hercules, CA, USA). GADPH was used as the internal control. The forward and reverse sequence of primers for GAPDH and BMP-2 are shown in Table 1. Real-time PCR and data analysis were performed using iQ5 Real Time PCR Detection System (Bio-Rad, Hercules, CA, USA). The cycling conditions were as the following: cDNA synthesis for 10 min at 50 °C; reverse transcription inactivation for 5 min at 95 °C; PCR amplification for 45 cycles with 10 s at 95 °C and 30 s at 60 °C. Melt curve analysis was performed as the following: 1 min at 95 °C, 1 minute at 55 °C and 80 cycles of 10 s at 55–95 °C. Expression of BMP-2 will be normalized to GADPH and 2 − ΔCt values will be calculated.

### 2.6. Statistical Analysis

Statistical analysis was performed using Statistical Package for Social Sciences version 20.0 (IBM, Armonk, NY, USA). Normality of the data was assessed using Shapiro-Wilks test. All data were normally distributed. Comparison of mean among the study groups were performed using one-way analysis of variance (ANOVA) with suitable post-hoc test. Additionally, the data were analyzed using factorial ANOVA considering the effects of lovastatin and annatto tocotrienol separately and together on each parameter. Statistical significance was defined as *p* < 0.05. The data were presented as mean ± standard error of mean. 

## 3. Results

**Figure 1:**
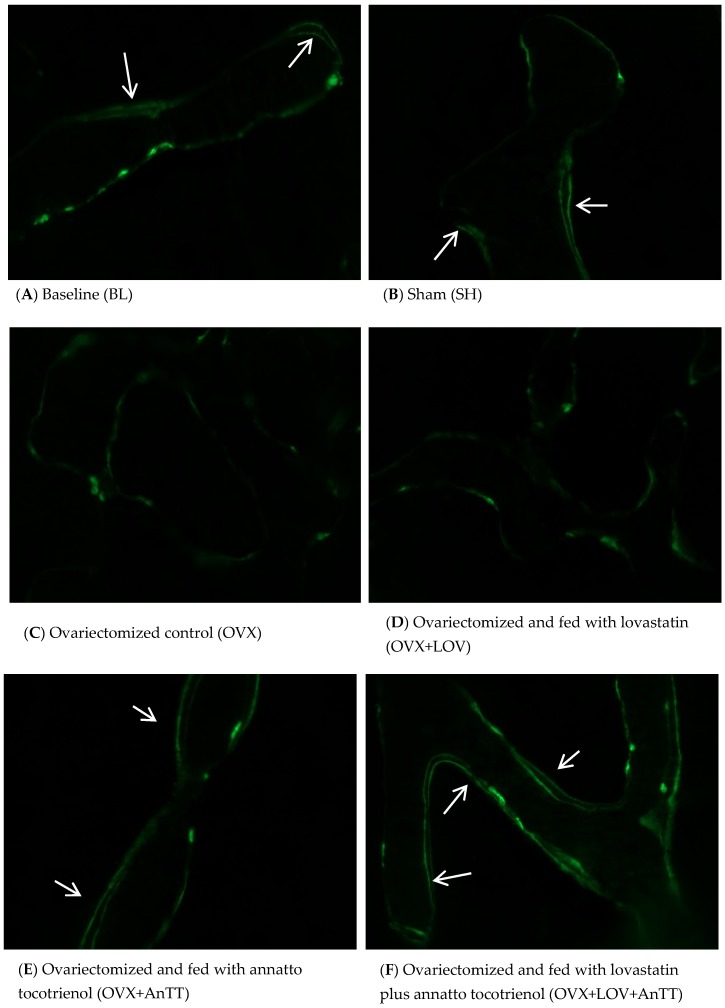
Micrograph of calcein-labeled trabecular bone. Rats treated with annatto tocotrienol alone or in combination with statin showed more calcein double-labeled surface. The white arrows show double-labeled surface.

**Figure 2:**
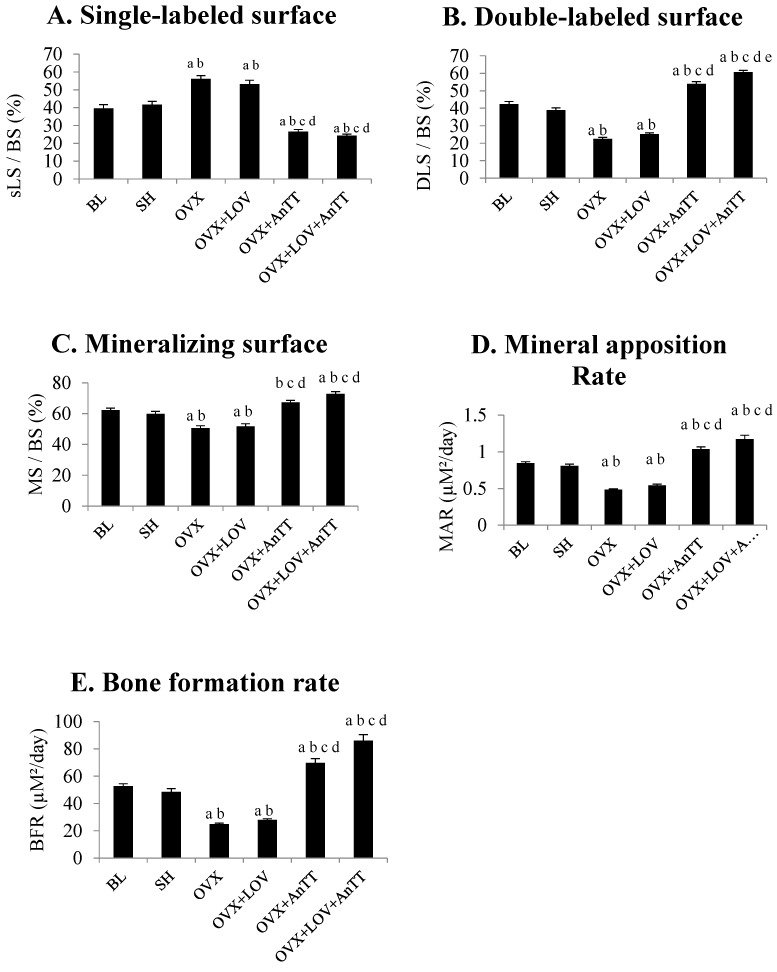
Bone dynamic histomorphometric parameters among the study group. Legend: Letters indicates significant difference between the marked group and ‘a’ BL; ‘b’ SH; ‘c’ OVX+LOV’; ‘d’ OVX+AnTT or ‘e’ OVX+LOV+AnTT. Abbreviation: BL = baseline; SH = sham-operated; OVX = ovariectomized; OVX+LOV = ovariectomized and supplemented with lovastatin (11 mg/day); OVX+AnTT = ovariectomized and supplemented with annatto tocotrienol (60 mg/kg/day); OVX+LOVAnTT = ovariectomized and supplemented with lovastatin (11 mg/day) and annatto tocotrienol (60 mg/kg/day).

From the fluorescent micrographs, trabecular bone of the ovariectomized rats treated with annatto tocotrienol alone or lovastatin and annatto tocotrienol together showed more calcein double-labelled surface compared to untreated rats and rats treated with lovastatin alone (Figure 1). Quantification using a Weibel grid revealed that the sLS/BS was significantly higher (*p* < 0.001), but dLS/BS (*p* < 0.001), MS/BS (*p* = 0.006), MAR (*p* < 0.001), BFR (*p* < 0.001) were significant lower in the OVX group compared to the SH group. These parameters were not significantly different in OVX+LOV group compared to OVX group (*p* > 0.05). In contrast, OVX+AnTT and OVX+LOV+AnTT group possessed significantly lower sLS/BS, but higher dLS dLS/BS, MS/BS, MAR, and BFR compared to the OVX group (*p* < 0.001 for all comparisons). The sLS/BS (*p* = 0.935), MS/BS (*p* = 0.127), MAR (*p* = 0.458), and BFR (*p* = 0.175) between ovariectomized rats receiving combined treatment of annatto tocotrienol and lovastatin and those receiving annatto tocotrienol alone were not significantly different. Only the dLS/BS was significantly different between the two groups (*p* = 0.002) (Figure 2A–E). 

**Figure 3:**
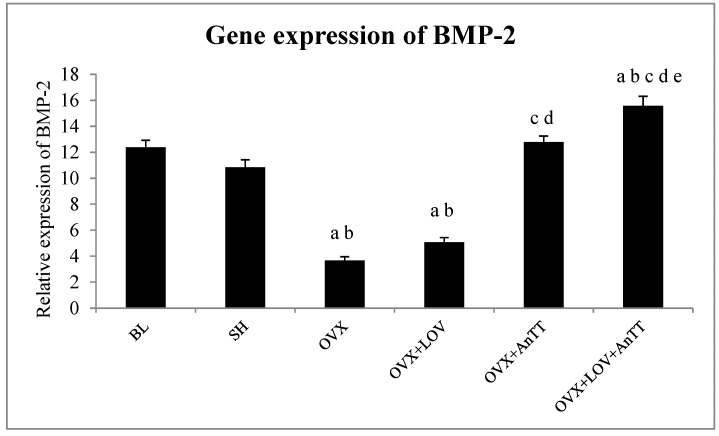
Gene expression of BMP-2 among the study groups. Legend: Letters indicates significant difference between the marked group and ‘a’ BL; ‘b’ SH; ‘c’ OVX+LOV’; ‘d’ OVX+AnTT or ‘e’ OVX+LOV+AnTT. Abbreviation: BL = baseline; SH = sham-operated; OVX = ovariectomized; OVX+LOV = ovariectomized and supplemented with lovastatin (11 mg/day); OVX+AnTT = ovariectomized and supplemented with annatto tocotrienol (60 mg/kg/day); OVX+LOVAnTT = ovariectomized and supplemented with lovastatin (11 mg/day) and annatto tocotrienol (60 mg/kg/day).

The relative expression of BMP-2 mRNA was significantly lower in the OVX group compared to the SH group (*p* < 0.001). Treatment with lovastatin did not elevate the expression of BMP-2 mRNA significantly compared to the OVX group (*p* = 0.409). Annatto alone (*p* < 0.001) or in combination with lovastatin (*p* < 0.001) significantly increased the expression of BMP-2 mRNA compared to the OVX group. The increase was significantly higher in the OVX+LOV+AnTT group compared to the OVX+AnTT group (*p* = 0.006) (Figure 3).

The data were analyzed again using factorial ANOVA to determine the individual and combined effects of lovastatin and annatto tocotrienol on each parameter. The main effect of lovastatin was significant for dLS/BS (*p* < 0.001), MAR (*p* = 0.006), BFR (*p* = 0.003), and BMP-2 (*p* < 0.001). The main effect of annatto tocotrienol was significant for all parameters studied (*p* for all parameters < 0.001). Significant interaction (lovastatin × annatto tocotrienol) was observed for dLS/BS (*p* < 0.001) and BFR (*p* = 0.037). These results indicated that the effects of annatto tocotrienol and lovastatin on MAR and BMP-2 could be additive, and on dLS/BS and BFR could be synergistic.

## 4. Discussion

The current study showed that co-supplementation of lovastatin and annatto tocotrienol was superior to lovastatin or tocotrienol alone in improving bone formation and mineralization activity in rats with estrogen deficiency, indicated by lower sLS/BS, but higher dLS/BS, MS/BS, MAR, and BFR compared to the untreated group. Annatto tocotrienol at 60 mg/kg body weight was able to improve bone dynamic histomorphometry of the ovariectomized rats. The combined treatment was more efficacious than annatto tocotrienol alone in increasing dLS/BS and BMP-2 expression. Lovastatin at the usual hypocholesterolemic dose in rats failed to augment the bone dynamic histomorphometry in ovariectomized rats within eight weeks. The skeletal anabolic effects of the aforementioned treatment regimens corresponded well to the increase in skeletal expression of BMP-2 of the rats (OVX+LOV+AnTT > OVX+AnTT = SH > OVX+LOV = OVX). The effects of annatto tocotrienol and lovastatin could be additive for MAR and BMP-2, and synergistic for dLS/BS and BFR. 

Considering the higher metabolic rate of rats, 10 mg/kg/day of statins to rats was equivalent to 70 mg/day in humans [34]. The dose of lovastatin administrated to rats in this study was 11 mg/kg/day, which was equivalent to 77 mg/day to human. Oral administration of lovastatin as low as 10 mg/kg was shown to reduce the serum cholesterol level in ovariectomized rats [35]. The lovastatin dose used in this study does not exceed the recommended statin dose for high-intensity hypocholesterolemic effects in humans (80 mg/day) [36]. Monteiro et al. showed that oral supplementation of very-high-dose simvastatin (20 mg/kg/day, equivalent to 140 mg/day in humans) improved the bone microstructure of ovariectomized rats in 14 days [37]. Similar effects were not observed with a lower dose within the same treatment period [37]. Thus, it is reasonable that lovastatin at the dose used in the current study produced no effects on bone dynamic histormorphometric parameters in ovariectomized rats. Similarly, a study showed that simvastatin at 10 mg/kg/day for five weeks did not exert bone anabolic effects in normal female rats [38]. Our previous studies also showed that lovastatin at 11 mg/kg did not improve bone microstructure and mechanical strength in ovariectomized rats [26,27]. Another study showed that simvastatin at 10 mg/kg/day could not reverse established osteoporosis in ovariectomized rats [39]. The lack of improvement in bone formation and mineralization in rats supplemented by statins at hypocholesterolemic dose, as illustrated in this study, provided an explanation for the aforementioned studies. Deposition of statins in skeletal tissue after oral administration of statins is very low [35], thus a lower dosage and short treatment period prevents statins from achieving their bone protective potential. 

Tocotrienol has been shown to promote bone mineralization and formation process in various animal studies [23,28,40,41]. Despite the difference in composition of tocotrienol homologues, bone dynamic histomorphometric changes caused by palm tocotrienol in ovariectomized rats were comparable with alterations induced by annatto tocotrienol observed in this study [23,40]. They were marked by a reduction in sLS/BS, and an increment in dLS/BS, MS/BS, MAR, and BFR in the supplemented ovariectomized rats compared to the untreated group [23,40]. The improvement in bone dynamic histomorphometry caused by palm tocotrienol was greater than estrogen treated group in a study by Aktifanus et al. [40]. In orchidectomized rats, annatto tocotrienol at 60 mg/kg/day for eight weeks caused a decrease in sLS/BS and an increase in dLS/BS, but the changes in MS/BS, MAR and BFR were not significant [28]. This might indicate that annatto tocotrienol works better in a female bone loss model. The rise in bone formation and mineralization caused by annatto tocotrienol could be explained by increased osteoblastic activity, marked by increased circulating bone formation markers and increased gene expression of osteoblastic differentiation markers [26,28,42]. It also corresponded to the previous findings that osteoblast number, osteoid surface, osteoid volume were inflated in ovariectomized rats supplemented with annatto or palm tocotrienol [22,26,42]. The dose of tocotrienol used in this study (60 mg/kg/day) is well below the toxic dose detected in previous animal studies [43]. 

The combination of tocotrienol and lovastatin was found to increase the dLS/BS and BMP-2 expression better than individual treatments. In addition, there were potential additive effects (MAR and BMP-2) and synergistic effects (dLS/BS and BFR) between annatto tocotrienol and lovastatin. This indicates that statin users could experience bone protection without increasing the dose of medication beyond the current recommendation. Previous studies have demonstrated that the combined treatment of tocotrienol and lovastatin enhanced the bone microstructure, increased osteoblast number and osteoid production, and decreased osteoclast number and bone erosion in ovariectomized rats better than both agents alone [26,27]. The rats treated with both agents concurrently also had significantly higher bone biomechanical strength compared to rats receiving single treatment of either agent [27]. Both tocotrienol and lovastatin are suppressors of the mevalonate pathway important for isoprenoid synthesis by inhibiting the rate-determining HMGCR enzyme via modulation of sterol regulatory element-binding proteins (SREBPs) [10,44]. These isoprenoids are materials for cholesterol synthesis or prenylation with GTPases to produce prenylated proteins, which act as negative regulators for bone formation [10,44]. Gamma- and delta-tocotrienol, the constituents of annatto tocotrienol mixture, modulate HMGCR in slightly different ways. Delta-tocotrienol enhances the ubiquitination of HMGCR and inhibits SREBP processing [45]. Gamma-tocotrienol is more selective in HMGCR degradation than blocking SREBPs [45]. Structurally, tocotrienols with their long carbon chain with double bonds are similar to farnesyl, a compound preceding geranyl-pyrophosphate that will enter isoprenoid synthesis [46]. The presence of tocotrienol stimulates the farnesol production instead of farnesyl, thus reducing the input for isoprenoid synthesis pathway [46]. On the other hand, statins are competitive inhibitor of HMGCR because they are structurally similar with HMGCo-A, the substrate for HMGCR [47]. The benefits of tocotrienol and statins co-treatment extend beyond bone health, and have been proven in anticancer studies [48,49]. 

Bone morphogenetic protein-2 plays an important role in the differentiation of osteoblasts. Through Smad signaling pathway, BMP-2 activates runt-related factor-2 (RUNX2), the master transcription factor for osteoblastic gene expression [50]. It can also activate osterix, an essential transcription factor for the differentiation of osteoblasts directly via distal-less homeobox 5 or indirectly via RUNX2 [50]. The expression of BMP-2 is influenced by the mevalonate pathway. Simvastatin treatment was shown to increase the expression of BMP-2 in preosteoblasts, decrease post-translation modification of Ras, regulate intracellular protein associated to Ras, and subsequently increase osteoblast differentiation [51]. Results of the current study showed that hypocholesterolemic dose of lovastatin could not upregulate the expression of BMP-2 in bone probably due to poor deposition of the compound in the skeleton. On the other hand, tocotrienol alone or in combination with lovastatin increased the expression of skeletal BMP-2. The extent of improvement was greater in the latter compared with the former, partly due to the additive effect of both agents. Previous studies have established that tocotrienol was able to preserve BMP-2, RUNX2, and OSX expression in nicotine-treated osteoporotic rats [52]. Tocotrienol was also shown to increase gene expression of osteoblast markers, such as ALPL, COL1A1, SPP1, and CTNNB1 in orchidectomized rats [28]. The current study showed that these changes could be a result of BMP-2 up-regulation since they are all downstream genes of BMP-2 signaling. 

Several limitations should be considered in this study. Only gene expression of BMP-2 was determined. Its expression level was not validated by protein expression assay. Tocotrienol has similar cholesterol-lowering effects as statins [53,54]. However, this study did not investigate whether concurrent treatment with tocotrienol would potentiate the hypocholesterolemic effects of statins. Despite strong evidence from previous studies, we could not validate the involvement of the mevalonate pathway in the bone protective action of both agents directly. This is because we did not quantify the inhibition of HMGCR and level of prenylated proteins in the bone. In spite of these limitations, this study successfully showed that tocotrienol could aggrandize the bone protective actions of low-dose statins by increasing bone mineralization and formation. This could protect middle-aged and elderly populations already taking statins for hypercholesteremia against osteoporosis. 

## 5. Conclusions

Tocotrienol alone or in combination with low-dose lovastatin can augment bone formation and mineralization in a rat model of bone loss due to estrogen deficiency. The enhanced protection can be contributed by the additive or synergistic effects between lovastatin and annatto tocotrienol on bone. The bone protective action of both regimens is mediated by an increased skeletal BMP-2 expression. This provides a justification to conduct a clinical trial supplementing tocotrienol in statins users to protect them against osteoporosis. 

## Figures and Tables

**Table 1:** Primers for GADPH and BMP-2.

Gene	Accession Number	Primer Sequence	Base Pairs
GAPDH	NM 017008	F: 5′-GTGGACCTCATGGCCTACAT-3′	129
		R: 5′-TGTGAGGGAGATGCTCAGTG-3′	
BMP-2	NM 017178	F: 5′-TGAACACAGCTGGTCTCAGG-3′	120
		R: 5′-TTAAGACGCTTCCGCTGTTT-3′	
